# Voltage-gated calcium channel blockers for psychiatric disorders: genomic reappraisal

**DOI:** 10.1192/bjp.2019.157

**Published:** 2020-05

**Authors:** Paul J. Harrison, Elizabeth M. Tunbridge, Annette C. Dolphin, Jeremy Hall

**Affiliations:** 1 Professor, Department of Psychiatry, University of Oxford; and Honorary Consultant Psychiatrist, Oxford Health NHS Foundation Trust, UK; 2 Associate Professor, Department of Psychiatry, University of Oxford; and Oxford Health NHS Foundation Trust, UK; 3 Professor, Department of Neuroscience, Physiology, and Pharmacology, University College London, UK; 4 Professor, Neuroscience and Mental Health Research Unit, Cardiff University, UK

**Keywords:** Calcium channels, therapy, psychiatry, bipolar affective disorders, novel central nervous system drugs

## Abstract

We reappraise the psychiatric potential of calcium channel blockers (CCBs). First, voltage-gated calcium channels are risk genes for several disorders. Second, use of CCBs is associated with altered psychiatric risks and outcomes. Third, research shows there is an opportunity for brain-selective CCBs, which are better suited to psychiatric indications.

Calcium channel blockers (CCBs) block voltage-gated calcium channels (VGCCs) and have been widely used to treat hypertension and other cardiovascular conditions for 40 years.^1^ Interest in the possibility that CCBs might also have value in psychiatry, especially bipolar disorder, goes back almost as far. This reflected several factors, including the critical roles of ionic calcium in neuronal function and neurotoxicity, its putative involvement in mood regulation, and the calcium-modulating effects of lithium. However, CCBs did not find an established position in the treatment of any psychiatric disorder, and interest in the topic had dwindled by the early 2000s.^2^ Nevertheless, developments over the past decade suggest that it is premature to dismiss VGCCs and CCBs as having no potential therapeutic value in psychiatry.^3^

## A primer on calcium channels and CCBs

**Fig. 1:**
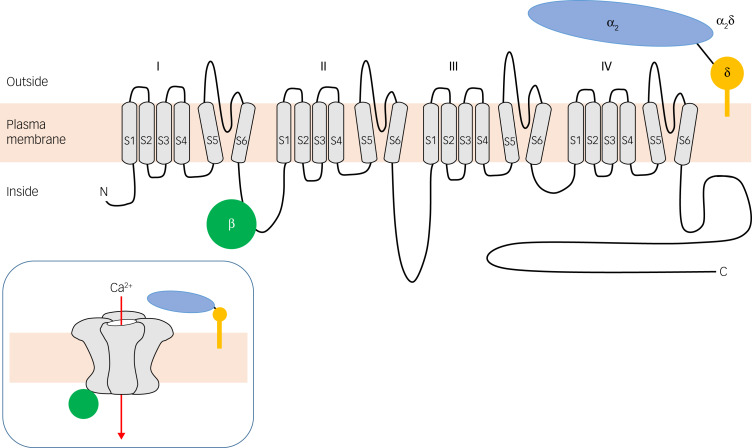
Voltage-gated calcium channel topology. The α1 subunit is a large transmembrane protein with intracellular amino (N) and carboxyl (C) termini. It has four voltage-sensing domains (I–IV), each of which spans the plasma membrane via six helices (S1–S6). The smaller β (green) and α2δ (blue/yellow) auxiliary subunits are also shown. The inset illustrates how the α1 subunit folds to produce the pore through which calcium ions pass when the membrane is depolarised.

VGCCs flux calcium across excitable cell membranes.^4^ As shown in Fig. 1, they comprise a pore-forming α_1_ subunit accompanied by β and usually α_2_δ auxiliary subunits; γ subunits are also described but are not in fact part of most VGCCs.^5^ VGCC nomenclature is complex (Table 1).^6^ The channels are grouped into ‘types’ based on their properties (e.g. L-type refers to large and long-lasting currents), which in turn are largely determined by the constituent α_1_ subunit. It is the L-type VGCCs that are the primary target of CCBs, with *CACNA1C* (Ca_V_1.2) and *CACNA1D* (Ca_V_1.3) being the main subtypes expressed in the brain, and hence of most psychiatric relevance.

**Table 1:** Voltage-gated calcium channel nomenclature and genetic associations to psychiatric disorders

Subunit type	Channel type	Drug target for	Subunit name	Channel name^a^	Gene symbol	GWAS association	Rare variants
Alpha (α1)	L-type	All CCBs	α1S	Ca_V_1.1	*CACNA1S*	XD	Scz
			α1C	Ca_V_1.2	*CACNA1C*	BD, Scz, ASD, XD	ASD, BD, Scz
			α1D	Ca_V_1.3	*CACNA1D*	XD	ASD, BD
			α1F	Ca_V_1.4	*CACNA1F*		
	P-/Q-type		α1A	Ca_V_2.1	*CACNA1A*		
	N-type		α1B	Ca_V_2.2	*CACNA1B*		BD, Scz
	R-type	Lamotrigine, topiramate	α1E	Ca_V_2.3	*CACNA1E*	MDD, XD	
	T-type	Some CCBs, e.g. nimodipine	α1G	Ca_V_3.1	*CACNA1G*		
			α1H	Ca_V_3.2	*CACNA1H*		ASD, Scz
			α1I	Ca_V_3.3	*CACNA1I*	ASD, Scz	
Beta (β)			β1		*CACNB1*		
			β2		*CACNB2*	Scz	ASD
			β3		*CACNB3*		
			β4		*CACNB4*		Scz
Alpha2delta (α2δ)		Pregabalin, gabapentin	α2δ1		*CACNA2D1*	MDD	Scz
		Pregabalin, gabapentin	α2δ2		*CACNA2D2*	XD	Scz
			α2δ3		*CACNA2D3*		ASD
			α2δ4		*CACNA2D4*	XD	Scz

GWAS, genome-wide association study; CCB, calcium channel blockers; XD, cross-diagnostic (schizophrenia/bipolar disorder/major depressive disorder/autism spectrum disorder/attention-deficit hyperactivity disorder); Scz, schizophrenia; BD, bipolar disorder; ASD, autism spectrum disorder; MDD, major depressive disorder.

a. Channel name defined by α1 subunit, so terminology does not apply to β or α2δ subunits.

CCBs are a structurally diverse class. The major subtypes are benzothiazepines (e.g. diltiazem), phenylalkylamines (e.g. verapamil) and dihydropyridines (DHPs, which comprise the other CCBs, including most in current use). All classes bind reversibly to the α_1_ subunit; verapamil binds in the pore itself, whereas DHPs bind to a nearby transmembrane domain to alter the shape of the pore and thereby prevent Ca^2+^ flow. Among DHPs, individual drugs show some differences, such as their relative preference for L-type VGCC subtype, their half-life and their permeability across the blood–brain barrier.^3^^,^^6^ All of these factors might affect their use in psychiatry.

## Recent VGCC and CCB developments

The most important stimulus reinvigorating psychiatric interest in CCBs was the discovery that VGCCs are part of the genetic risk architecture for a range of disorders. The initial report was of the association of a polymorphism within *CACNA1C* in an early genome-wide association study of bipolar disorder; the evidence has grown to encompass genome-wide significance for several VGCC subunits across a range of disorders (Table 1).^7^ There is also some evidence for disease-associated rare variants in some VGCCs, notably mutations in *CACNA1C* which cause Timothy syndrome, a disorder characterised by arrhythmias, syndactyly and autism.

The disease associations of VGCCs shown in Table 1 share all the caveats of other genome-wide association study findings, such as the very small effect size, the unidentified causal variants and the unknown molecular correlates of the risk alleles.^8^ Despite the uncertainties, VGCCs have the notable attraction that, in contrast to most implicated psychiatric genes, they are known to be ‘druggable’. This is not just because of CCBs but due to other licensed drugs such as gabapentin and pregabalin, which are ligands of the α_2_δ subunit, and lamotrigine and topiramate, which block VGCCs among other actions. Hence, VGCCs are the poster child for showing whether and how genomic discoveries can lead to novel psychiatric treatments, a promise often made but one which involves a long and largely uncharted path.^9^ Another implication of the genomic findings is that response to CCBs might be influenced by VGCC genotype, although this has not been tested other than in a pilot study.^10^

In addition to the statistical associations with psychiatric disorders, growing evidence shows that VGCCs contribute to relevant brain phenotypes. Studies with genetically modified rodents reveal that L-type VGCCs affect domains of cognition, mood, emotion and reward.^11^ Some effects are developmentally specific and accompanied by alterations in synaptic plasticity and neurogenesis.^12^^,^^13^ There is also increasing evidence that centrally acting CCBs affect similar processes, including forms of memory and plasticity.^14^ In humans, VGCC risk alleles are associated with a range of alterations in brain structure, task performance and in patterns of neural activation.^15^ However, the findings in both rodents and humans are varied, and their interpretation complex. As a result, although the literature provides increasingly strong support for a role of L-type VGCCs in many aspects of brain and behaviour, the details and implications remain unclear.

An obvious counter to the genomically driven proposal that VGCCs may be good psychiatric drug targets is that, as noted above, CCBs have already been tested and found largely ineffective in several disorders. However, most of the studies used the first generation of drugs, especially verapamil.^2^^,^^3^ Compared to the DHPs, these drugs are less selective and have poorer or unknown permeability across the blood–brain barrier. Thus it can be argued that CCBs have not been critically tested for psychiatric indications. Moreover, the studies were limited in terms of sophistication, breadth and scale. In particular, pursuing the genomic logic, since VGCCs contribute to a range of disorders, CCBs may have value in treating transdiagnostic features such as mood instability, cognitive dysfunction and circadian disruption. To address these possibilities, Atkinson and colleagues^16^ are investigating the effect of nicardipine – a brain-penetrant DHP – on mood, sleep and cognition. The study also assesses nicardipine's effects on brain activity, using functional MRI and magnetoencephalography, as well as leucocyte calcium channel expression and calcium flux.

The extensive cardiovascular prescribing of CCBs allows for pharmacoepidemiological studies to assess whether their use is associated with an altered risk or course of neuropsychiatric disorders. Hayes *et al*^17^ recently showed that, among patients with psychosis, rates of psychiatric admission and self-harm were 10–20% lower during periods of CCB treatment. The design rules out confounding by indication and no similar benefits were seen for thiazide diuretics or for non-psychiatric admissions, suggesting a degree of specificity. There is also meta-analytic evidence that CCB use is associated with 20–30% lower risks of developing dementia and Parkinson's disease.^18^ However, there are also preliminary data suggesting higher admission rates for depression in people taking CCBs,^19^ a finding requiring further evaluation. Nevertheless, the epidemiological data tentatively suggest that CCBs may have beneficial preventative or ameliorating effects on a range of neuropsychiatric disorders. It is unclear whether the postulated benefits are mediated directly via central VGCC antagonism or indirectly via cardiovascular effects or other mechanisms, such as lowering oxidative stress or inducing autophagy.^20^ Regardless, the data provide encouragement to conduct further psychiatric clinical trials of CCBs.

## Targeting brain VGCCs

Even if some psychiatric benefits of existing CCBs have been overlooked, these drugs are unlikely to be suitable, let alone optimal, agents for use, in part because of their cardiovascular effects and side-effects (e.g. headache, ankle swelling). A key advance would be CCBs that potently target brain VGCCs but spare those in the periphery. An opportunity to achieve this goal is provided by the fact that VGCC genes, including those encoding the α1 subunits, are expressed as multiple variants (‘isoforms’). Critically, some isoforms are more highly expressed in the brain than in the heart and they differ in some physiological and pharmacological properties.^6^^,^^18^ Until recently, the psychiatric relevance of these data was limited. First, they pertained to rodents and could not confidently be extrapolated to humans. Second, most isoforms were simply based on the inclusion or exclusion of individual exons; given that some VGCC genes contain over 50 exons, it was difficult to determine the structure of full-length transcripts or, therefore, the full-length protein. Third, many additional isoforms likely existed but had not been detected by the available methodologies.

These limitations can now be overcome utilising new technologies. By using long-range polymerase chain reaction and Nanopore sequencing, Clark *et al*^21^ found that *CACNA1C* is expressed in human brain as multiple messenger RNA isoforms, most of which had not been reported (in humans or in rodents), and with several being abundant and affecting key functional domains of the channel. The experimental design strongly implies that the variants are all much more abundant in the brain than in the heart. This emerging evidence supports the existence of brain-enriched VGCC isoforms, providing targets for novel CCBs with the potential for reduced peripheral effects. Assuming the novel drugs were also penetrant of the blood–brain barrier, they would finally allow the value of CCBs for psychiatric indications to be tested critically.

## Conclusions

Until positive results from randomised clinical trials are reported, CCBs cannot be considered to have any role in psychiatry. However, the recent developments cumulatively and markedly strengthen the candidacy of VGCCs as therapeutic targets and encourage the development of drugs with selectivity for brain-enriched isoforms. As noted, it is primarily the genomic findings that have rekindled psychiatric interest in VGCCs. By the same token, it will be of considerable interest to see how the possibility that they are therapeutic targets develops: success would serve as an important proof of principle that psychiatric genomics can pay off therapeutically. Failure would still be valuable if lessons are learned regarding the strategies and approaches that work versus those that do not. The only unqualified failure for psychiatry will be if the science is done inadequately, or not done at all.
